# Antipsychotics and Risks of Cardiovascular and Cerebrovascular Diseases and Mortality in Dwelling Community Older Adults

**DOI:** 10.3390/ph17020178

**Published:** 2024-01-30

**Authors:** Sylvie Perreault, Laurie-Anne Boivin Proulx, Judith Brouillette, Stéphanie Jarry, Marc Dorais

**Affiliations:** 1 Faculty of Pharmacy, Université de Montréal, Montreal, QC H3C 3J7, Canada; 2 Centre de Recherche en Santé Publique (CReSP), Partenaire CIUSSS du Centre-Sud-de-l’Île-de-Montréal et l’Université de Montréal, Montreal, QC H3L 1M3, Canada; 3 Department of Cardiology, Faculty of Medicine, University of Ottawa Heart Institute, Ottawa, ON K1Y 4W7, Canada; laurieanneboivinproulx@gmail.com; 4 Department of Psychiatry and Addictology, Université de Montréal, Montreal, QC H3L 1M3, Canada; judith.brouillette@icm-mhi.org; 5 Department of Anesthesiology, Montreal Heart Institute, Université de Montréal, Montreal, QC H3L 1M3, Canada; stephanie.jarry.1@umontreal.ca; 6 StatSciences Inc., Notre-Dame-de-l’Île-Perrot, QC H3L 1M3, Canada; marc.dorais.statsciences@gmail.com

**Keywords:** antipsychotics, risk of cardiovascular diseases, cerebrovascular diseases and mortality

## Abstract

This study aims to investigate the effect of antipsychotic agents on cardiovascular and cerebrovascular diseases (CVD/CEV) and mortality risks in the older population living in a community. A cohort of 42,650 new users of antipsychotic agents was built using Quebec healthcare databases (1998–2011). The outcomes were CVD/CEV and mortality incidence in 5 years of follow-up in the total cohort, sub-cohort of patients with no schizophrenia/dementia, sub-cohort with schizophrenia, and sub-cohort with dementia. Comparisons were made between the new users who continued the treatment (adherent level ≥ 60%) vs. those ceasing treatment (adherence level < 60%) using inverse probability of treatment weighting and Cox models. Comparing high adherence vs. low levels, CVD/CEV risk was increased by 36% in the sub-cohort with schizophrenia for atypical antipsychotic users and by 25% in the sub-cohort with dementia for typical antipsychotic users. An increasing mortality risk of 2- to 3-fold was linked with the typical antipsychotic use in all cohorts except the sub-cohort with schizophrenia; in addition, mortality risk is linked with the use of high vs. low doses. Antipsychotics were not linked with CVD/CEV risk, except for atypical antipsychotics in patients with schizophrenia and typical antipsychotics in patients with dementia. The mortality risk was linked with the use of typical antipsychotics and the dose used.

## 1. Introduction

The prevalence of antipsychotic use has increased by more than 200% in the last decades [1]. The use of antipsychotic agents has been linked with metabolic and neurologic side effects, as well as an increased mortality risk in older adults [2,3,4]. Despite concerns for the side effects and increased mortality risk, antipsychotic agents are often used for behavioral and psychological symptoms associated with dementia [5,6,7,8,9,10]. A meta-analysis of randomized controlled trials (RCT) published by Schneider et al. showed that atypical antipsychotic agents compared to placebo may present a small increase in mortality risk for patients with dementia [11]. A recent network meta-analysis of placebo-controlled RCTs with aripiprazole, risperidone, quetiapine, and olanzapine use showed no significant difference between these drugs and the slight improvement of clinical effectiveness, as well as the stroke and death risks in dementia [12].

Cardiovascular diseases (CVDs) are the leading causes of mortality in the world and have a substantial impact on morbidity in older adults [13,14]. The effect of antipsychotics on the risk of stroke, myocardial infarction (MI), and mortality among antipsychotic users and whether their impact differs in different older adult populations remains unclear [15,16,17,18]. Moreover, older adults are also at higher risk of multiple associated morbidities, polypharmacy, inappropriate prescribing, and physiological changes that can modify the pharmacodynamics and pharmacokinetics of drugs [19]. These factors expose older adults to an additional increasing risk of CVD, stroke, and mortality [16,17,20,21]. There is a need to clarify the cardiovascular disease and cerebrovascular disease (CVD/CEV) and mortality risks in different older adult populations, accounting for individual factors, duration of exposure, class of antipsychotic drugs, dose used, and comorbidities [16,17]. Given the limited and inconsistent evidence on CVD/CEV and mortality risks in older adults of different populations, we assessed the impact of the new use of antipsychotics in patients with behavioral and psychological symptoms, comparing the patients who continued the treatment vs. those ceasing treatment on CVD/CEV and mortality risks in several populations of older adults living in a community, accounting for individual factors, level of drug adherence, class of antipsychotics, dose used, and comorbidities.

## 2. Results

**Figure 1:**
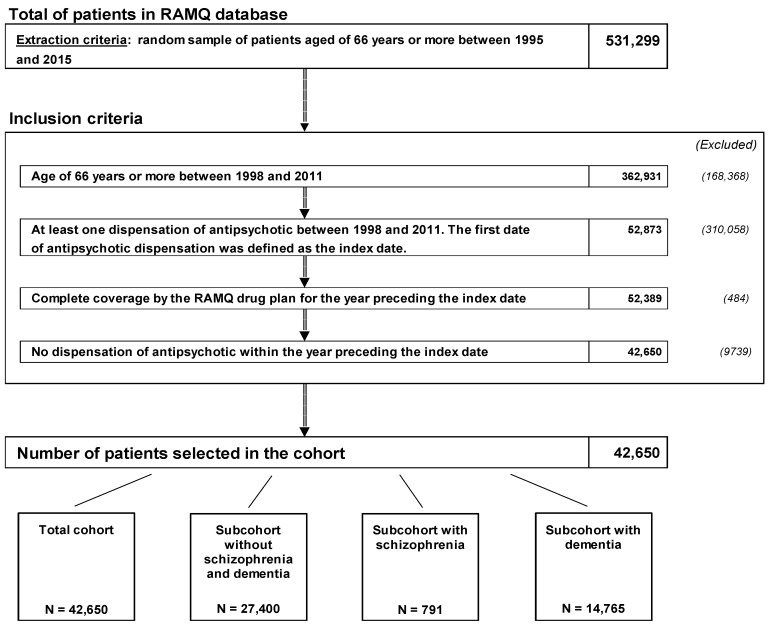
Study flow chart (RAMQ, Régie d’Assurance Maladie du Québec).

A total of 42,650 subjects were included as new users of antipsychotics (Figure 1). Patients’ characteristics are summarized in Table 1 for the total cohort, and those for the sub-cohorts are shown in Tables S1–S3. Baseline demographics and clinical characteristics of patients with an adherence level of <60% are quite similar to those with an adherence level of ≥60% for the total cohort and the sub-cohorts of new users of antipsychotics, and they were well balanced after an inverse probability of treatment weighting (IPTW).

### 2.1. Antipsychotics Exposure

The profile of use at the time of initiation and the mean equivalent olanzapine dose at the initiation and at 1-year of follow-up are shown in Table S4. At the initiation, atypical antipsychotic agents were used at 64.4%, 51.7%, 88.2%, and 87.3% among the total cohort, sub-cohort without schizophrenia/dementia, sub-cohort with schizophrenia, and sub-cohort with dementia, respectively. The mean olanzapine equivalent dose at the initiation ranged from 0.8 mg to 3.4 mg for atypical antipsychotics and was 10.2 mg for typical antipsychotics, which were mainly represented by haloperidol, prochlorperazine, and methotrimeprazine. Similar data were observed at 1-year of follow-up for atypical antipsychotics, except for typical antipsychotics (Table S4).

The mean (SD) of levels of adherence (%) in the total cohort were 16.2 (17.4) and 90.4 (11.5) using adherence levels of <60% and ≥60%, respectively; the median (Q1–Q3) were 8.5 (1.8–27.6) and 95.6 (83.7–100.0), respectively. Levels of adherence in the 5-year period of follow-up in the total cohort are shown in Table S5.

### 2.2. Cumulative Incidence of CVD/CEV and Mortality

**Figure 2:**
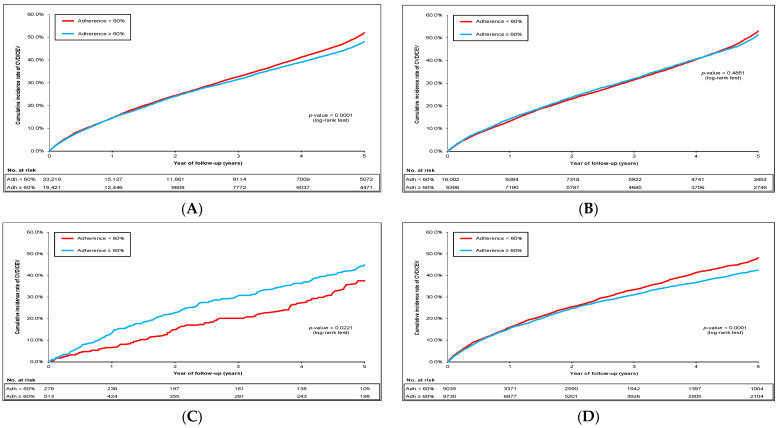
Cumulative incidence rates of CVD/CEV events after IPTW according to the adherence level (**A**) in the total cohort; (**B**) in the sub-cohort without schizophrenia/dementia; (**C**) in the sub-cohort with schizophrenia (*p*-value at 12 months: 0.0088); (**D**) in the sub-cohort with dementia.

The mean (SD) years of follow-up of the total cohort was 3.0 (2.0), with the median (Q_1_–Q_3_) of 3.2 (0.9–5.0). Figure 2A–D shows the cumulative incidence rates of CVD/CEV in the total cohort, sub-cohort without schizophrenia/dementia, sub-cohort with schizophrenia, and sub-cohort with dementia, respectively. The specific incident rates of CVD/CEV and mortality according to the adherence of ≥60% vs. <60% are shown in Tables S6–S8. The incident rates of CVD/CEV for all antipsychotic users ranged from 8.8 to 14.7 per 100 person-years in the total cohort and sub-cohorts (Table S6). Similar values of CVD/CEV incident rates were observed in the total cohort and sub-cohorts for atypical and typical antipsychotic users (Tables S7 and S8).

**Figure 3:**
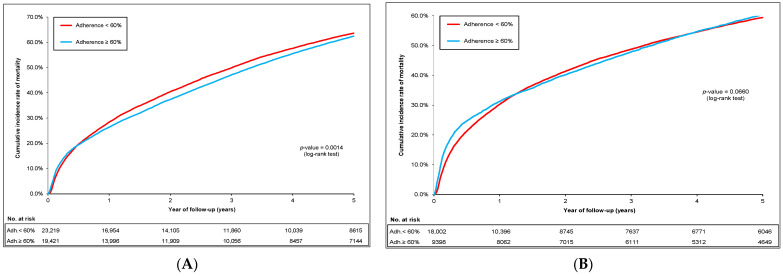

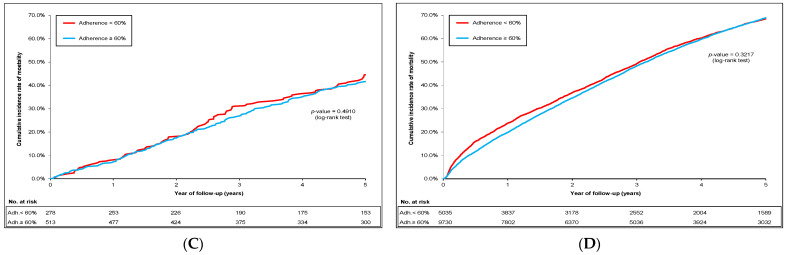
Cumulative incidence rates of mortality after IPTW according to adherence level (**A**) in the total cohort; (**B**) in the sub-cohort without schizophrenia/dementia; (**C**) in the sub-cohort with schizophrenia (*p*-value at 12 months: 0.0025); (**D**) in the sub-cohort with dementia.

Figure 3A–D shows the cumulative mortality rates in the total cohort, sub-cohort of patients without schizophrenia/dementia, sub-cohort with schizophrenia, and sub-cohort with dementia, respectively. The mortality rates in the 5-year period ranged from 20.1 to 23.4 per 100 person-years for all antipsychotic users in the total cohort, sub-cohort without schizophrenia/dementia, and sub-cohort with dementia, except for the sub-cohort with schizophrenia, ranging from 10.6 to 11.5 per 100 person-years (Table S8). However, the mortality rates changed substantially among adherent patients using typical antipsychotics, which ranged from 55.5 to 82.0 per 100 person-years in the total cohort, sub-cohort without schizophrenia/dementia, and sub-cohort with dementia (Table S8), but not in the sub-cohort with schizophrenia. In contrast to typical antipsychotic users, we did not note any change in the mortality rate in atypical antipsychotic users (Table S7).

### 2.3. Hazard Ratios for CVD/CEV Risks

As shown in Table 2, patients with high vs. low adherence levels (reference) for all *antipsychotic users* presented a slightly lower CVD/CEV risk (hazard ratio (Hs) 0.92; 95% CI 0.89–0.96) in the total cohort, sub-cohort without schizophrenia/dementia (HR 0.94; 95% CI 0.90–0.99), and sub-cohort with dementia (HR 0.89; 95% CI 0.83–0.94), which seems to be related to CAD and/or stroke events (Table S6). However, the CVD/CEV risk was increased by 37% in the high vs. low adherence levels (HR 1.37; 95% CI 1.06–1.77) in the sub-cohort with schizophrenia, where time-to-event was shortly identified following the drug initiation achieving the significance level at 12 months (Figure 2C).

Among *atypical antipsychotic* users, the high adherence level vs. low level was related to a slightly lower CVD/CEV risk (HR 0.93; 95% CI 0.90–0.96) in the total cohort and in the sub-cohort with dementia (HR 0.87; 95% CI 0.84–0.91), which seems to be related to CAD and/or stroke events (Table S7). Although, in the sub-cohort with schizophrenia, the CVD/CEV risk was increased by 33% in the high adherence level vs. low level (HR 1.33; 95% CI 1.04–1.70).

Among *typical antipsychotic* users with high vs. low adherence levels, no increased CVD/CEV risk was noted in the total cohort (HR 0.96; 95% CI 0.87–1.06) and in the sub-cohort with schizophrenia (HR 0.89; 95% CI 0.49–1.62) (Table 2). The CVD/CEV risk in the sub-cohort without schizophrenia/dementia was decreased by 21% with high vs. low adherence levels (HR 0.79; 95% CI 0.69–0.91), which seems to be linked to stroke (Table S8). The CVD/CEV risk was increased by 25% in high vs. low adherence levels in the sub-cohort with dementia (HR 1.25; 95% CI 1.04–1.51), where the rate of CAD and MI was significantly higher (Table S8).

### 2.4. Hazard Ratios for Mortality Risks

Among all antipsychotic users, high vs. low adherence levels presented a decrease in mortality risk in the total cohort (HR 0.96; 95% CI 0.94–0.98). There was no difference in mortality risk in the sub-cohort without schizophrenia/dementia (HR 0.98; 95% CI 0.94–1.01), sub-cohort with schizophrenia (HR 0.92; 95% CI 0.74–1.15), and sub-cohort with dementia (HR 0.98; 95% CI 0.94–1.02).

However, the mortality risk was significantly increased in *typical antipsychotic* users in the total cohort, sub-cohort without schizophrenia/dementia, and sub-cohort with dementia, where the patients with high vs. low adherence levels were associated with 2.49- to 3.55-fold increased mortality risk. On the other hand, we did not notice any increase in mortality risk in the patients with high vs. low adherence levels among *atypical antipsychotic* users. When exploring the impact of being users of high dose vs. low dose of *typical antipsychotics* (Table 3), the mortality risk was significantly increased by 1.67, 1.64, and 1.69 in the total cohort, sub-cohort without schizophrenia/dementia, and sub-cohort with dementia, respectively.

### 2.5. Sensitivity Analyses

First, analyses were repeated with a different threshold to distinguish high and low adherence groups. HRs for the CVD/CEV and mortality risk analyses using a level of adherence ≥ 70% vs. <70% were well aligned with the estimates of ≥60% vs. <60% (Table S9). Second, the rate per 100 person-years of hospitalization for glaucoma and hyperthyroidism, as a negative control, was similar among patients with high vs. low adherence levels (Table 4). Third, the E-value closest to boundary one for the mortality risk in the total cohort was 3.55; hence, the HR that this mortality risk could be explained by an unmeasured confounder occurred 6.24 times more frequently in patients with high vs. low adherence levels (Table 5). The high E-values indicate that the statistically significant results are robust with regard to unmeasured confounding factors.

## 3. Discussion

This study suggested that, in older populations living in a community, antipsychotic use did not seem to be linked with an overall increased CVD/CEV risk and mortality, but rather a decrease in both outcomes, except for high levels of adherence that were associated with a 33% increase in CVD/CEV outcomes among atypical antipsychotic users in the sub-cohort with schizophrenia and with a 25% increase in the CVD/CEV risk for typical antipsychotic users in the sub-cohort with dementia. Again, the mortality risks were increased by 2 to 3 folds among high adherence levels of typical antipsychotic users in all cohorts except for the sub-cohort with schizophrenia; in addition, these mortality risks seem to be linked to the use of high vs. low dose typical antipsychotics.

These results contrast and add to the current mixed literature in showing that this interaction is complex and sensitive to the population or sub-group studied. Our population consisted of older adults who still lived in a community and may be healthier than those of studies done in patients in long-term facilities [4]. Furthermore, we compared the CVD/CEV outcomes and mortality between adherent and non-adherent groups, which control for the underlying conditions for which patients were prescribed antipsychotics. Finally, we used an IPTW approach to account for differences in patient characteristics between adherent and non-adherent groups, which may differentiate us from other studies that did not. Our results also suggest that when antipsychotics are used in indications outside schizophrenia/dementia, higher adherence is associated with lower CVD/CEV outcomes. One hypothesis may be that treating insomnia, depression, or anxiety may be associated directly, or indirectly, toward a healthier lifestyle and adherence to CVD/CEV drugs to lower CVD/CEV risk. Indeed, is it known that the prevalence of comorbid psychological distress, including symptoms of depression, anxiety, and/or stress, is two to three times higher among chronic physical diseases than among healthy individuals [22]. It is also associated with non-adherence to treatment and self-management recommendations [23], poor lifestyle decisions [24], and poorer prognosis and early mortality in patients with chronic diseases [25,26], such as CAD [27,28].

Many factors may contribute to explaining the dose-dependent link between antipsychotic use and an increase in CVD/CEV risk in older adults. First, they are more likely to have several morbidities, leading to an increased risk of polypharmacy. Age-related physiologic changes in cardiovascular, gastric, hepatic, and renal function can also lead to changes in the pharmacokinetics of medications and drug interactions [29]. Second, antipsychotic use has been associated with weight gain and metabolic syndrome through different affinities for histamine, serotonin, and muscarinic receptors [30]. Antipsychotic medication also increases the risk of thrombosis. A meta-analysis reported that antipsychotic use was linked with an increased risk of venous thrombosis with major heterogeneity in the potential mechanisms including increased platelet aggregation and the somnolence that could lead to venous stasis [31]. Antipsychotic agents can increase insulin resistance, which is linked with cardiovascular and cerebrovascular disease [32]. Corrected QT interval (QTc)-prolonging antipsychotics can also increase the risk of sudden cardiac death [15,33].

Based on a recent systematic review and meta-analysis of observational studies, the effect of antipsychotic agents on the risk of stroke and MI versus not being users remained unclear [16]. They reported that (i) antipsychotic medication use seems to be linked with an increasing risk of stroke by 2-fold in the general population but with a substantial heterogeneity (I^2^ = 83.2%), compared to non-user patients [16]; (ii) there was no clear link established between the risk of stroke and psychiatric disorders (defined as schizophrenia, bipolar disorder, major depression, or dementia) in a population with a high level of heterogeneity (I^2^ = 78.8%); and (iii) the risk of stroke in patients with dementia was much lower (pooled HR: 1.16; 1.00–1.33) without heterogeneity. Concerning the MI risk and being users of antipsychotics vs. non-user patients, there is no clear evidence. This systematic review and meta-analysis were heterogeneous in terms of study design, antipsychotic drug type, study population, measure of effect, and the presence of substantial statistical heterogeneity, making it difficult to draw conclusions [16].

However, data on the impact of antipsychotic use and the risk of stroke in older adults using self-controlled designs reported an association with an increase in stroke risk shortly after the initiation [34,35]. The study of Fife et al. [36] compared the stroke risk in new typical vs. atypical antipsychotic users in patients aged over 65 years, using an approach of on-treatment after an adapted propensity score match 1:10 and Cox model [36]. Their participants had received a claim of antipsychotics and had no diagnosis of stroke in at least 183 days prior to the initiation. The study results reported that typical vs. atypical antipsychotics increased the stroke risk by 31% (HR: 1.31; 1.07–1.60) and that the risk was 1.45 (HR: 1.45; 1.17–1.80)-fold higher for haloperidol vs. atypical antipsychotics. Those results differ from our results since we are not using the same population, comparators, and primary outcomes.

There is a limited amount of literature about the comparative mortality risk and antipsychotic use among older adults living in the community. The study by Gerhard et al., 2014 [17] assessed the comparative mortality risk of antipsychotic new users in a community of older adults and suggested that the choice and dose of antipsychotics impact the mortality risk. For instance, the study reported an increased mortality risk of 18% for haloperidol vs. risperidone and a protective effect of 19% for quetiapine and 18% for olanzapine vs. risperidone [17]. The study by Sutana et al., 2019, reported that typical antipsychotic new users present a higher mortality risk vs. atypical antipsychotic users in older adults with cardiovascular and cerebrovascular disease and having drug–drug interactions (HR: 1.33; 1.27–1.39); [37] and, it seems that having a score drug–drug interaction higher than one was linked with an increased mortality risk [37]. Lastly, recent results of a case-control study, from the European Association for the Study of diabetes 2023, reported that people with diabetes type 2 with CVD, albuminuria, heart failure, and QTc-prolonging medication use are related to sudden cardiac arrest, while among patients with diabetes type 2 without CVD history, low fasting glucose, severe hypertension, dyslipidemia, and the use of QTc-prolonging antibiotic, antipsychotic, and prokinetic medication are linked with sudden cardiac arrest [38].

Our study is one of the largest to examine the effect of antipsychotic agents on the incidence of CVD/CEV and mortality in older adults living in the community. The strengths and limitations are greatly related to the nature of the Quebec RAMQ administrative databases. As there is a single-payer public health care system in Quebec (Canada), Quebec RAMQ administrative database includes all individuals over 65 years old, limiting selection bias, and capturing all diagnoses, procedures, and drugs. We used an IPTW approach to minimize confounding biases, and we accounted for drug exposure, class of antipsychotics, and equivalent doses used. We also provided several sensitivity analyses and negative control.

Several limitations must be taken into consideration. First, administrative claims data depend on the exhaustive, accurate recording and coding of diagnoses, procedures, and drugs. Second, the observational study of administrative data might have been subject to confounding bias by unadjusted factors (e.g., autoimmune diseases, pro-inflammatory conditions, smoking, sedentary lifestyle, major depressive disorder, delirium, etc.). As prochlorperazine, a typical antipsychotic, is used to treat other symptoms than those of schizophrenia or dementia, such as nausea and vomiting, we cannot exclude that another associated latent or incident condition may have been unadjusted. However, we did control for malign cancer three years prior to entry and the number of hospitalizations one year prior to entry. Again, in the model of exploring the impact of doses used on mortality risk in total cohorts and sub-cohorts, we also adjusted for being users of prochlorperazine. Third, administrative claims data depend on the exhaustive, accurate recording and coding of diagnoses and procedures. Fourth, the accurate starting for CVD/CEV risk factors and diseases may not always be accurately recorded in administrative claims data, so the duration of the CVD/CEV disorders could not be accounted for, but we have used a 3-year period to assess risk CVD/CEV risk factors or diseases. Fifth, no distinction between types of dementia was performed, as the type of dementia is not reliably specified in administrative claims data. Sixth, quantitative measurements of blood pressure, glucose, and cholesterol were not available, and we could not take appropriate control of hypertension, diabetes mellitus, and cholesterol into account. Seventh, we could not adjust for factors such as economic, health, lifestyle, and education that could also be linked with CVD/CEV and mortality [39]. Eight, the excess CVD and mortality risks in people with major mental illness are probably multifactorial and could include poor lifestyle behaviors, increased risk of diabetes, shared genetic factors, and direct effects of physiological effects of mental illness. Nine, we could not exclude the relationship between morbidity and the risk of mortality. However, we used an IPTW approach to account for differences in patient characteristics and associated morbidities between adherent and non-adherent groups in the three years prior to the cohort entry minimizes the impact of confounding biases in observational studies. Again, the difference in the incident rates of CVD/CEV among adherent groups vs. non-adherent groups of typical antipsychotic users during follow-up (Table S8) could never explain the substantial increase in the mortality rates among adherent groups of typical antipsychotic users in the total cohort, sub-cohort without schizophrenia/dementia, and sub-cohort with dementia. Lastly, a lack of statistical power may be presented in the sub-cohort with schizophrenia.

## 4. Methods

### 4.1. Data Source and Ethics Declarations

We built a cohort study with data from the Quebec government’s administrative database of hospital discharges (Med-Echo) as well as the databases of the Quebec medical services and public drug plan, both administered by Régie de l’Assurance Maladie du Quebec (RAMQ). We linked the Med-Echo and RAMQ medical and public drug plan databases through encrypted provincial health insurance numbers. Combining the information from Med-Echo databases, RAMQ medical services, and RAMQ drug plan databases, we obtained a complete history of hospital mortality and hospital admissions, medical services, and drug use of patients in Quebec (Table S10) [40,41,42,43,44,45].

Data access requests for the RAMQ databases are approved by the *Commission d’accès à l’information du Québec*. All efforts were made to maintain data confidentiality. Information from these databases provided a comprehensive picture of hospitalizations. The protocol received the approval of the University of Montreal Ethics Committee appointed *Comité d’éthique de la recherche en sciences et en santé,* and all methods were performed in accordance with the relevant guidelines and regulations. The RAMQ covers all Quebec residents for the cost of physician visits, hospitalizations, and procedures, as well as 94% of Quebec citizens aged 65 and older for the drug insurance plan [46].

### 4.2. Cohort Definition

The cohort was based on a random sample of 40% of the total cohort of patients aged 66 years and above between January 1995 and December 2015 in the province of Quebec, Canada. From those, we selected patients who received a dispensation of antipsychotics between 1998 and 2011. The antipsychotic agents were composed of typical (chlorpromazine, flupentixol, fluphenazine, haloperidol, methotrimeprazine, perphenazine, pimozide, pipotiazine, prochlorperazine, thioridazine, thiothixene, trifluoperazine, and zuclopenthixol) and atypical antipsychotics (aripiprazole, clozapine, loxapine, lurasidone, olanzapine, quetiapine, risperidone, and ziprasidone). The first date of antipsychotic dispensation was defined as the index date. In order to identify new users, patients who received antipsychotics in the year preceding the index date were excluded.

In addition to the total cohort, we defined three sub-cohorts. A sub-cohort was composed of patients without schizophrenia/dementia diagnosis (ICD-9 or ICD-10) within a 3-year period prior to the index date. A sub-cohort was composed of patients having a diagnosis of schizophrenia (ICD-9 or ICD-10) within a 3-year period prior to the index date, and a sub-cohort was composed of patients having a diagnosis of dementia within a 3-year period prior to the index date (ICD-9 or ICD10 code or medication; see Table S11).

### 4.3. Adherence Level of Antipsychotics

Adherence to antipsychotic agents was evaluated using the medication possession ratio (MPR), which is the proportion of days medication was supplied over a given follow-up period, expressed as a percentage. The MPR of ≥60% was employed to compare adherent to non-adherent patients (<60%). We ran a sensitivity analysis by using an MPR of ≥70% vs. <70%.

### 4.4. Outcomes

The main outcomes were the occurrence of incident CVD/CEV and mortality events during the 5-year period of follow-up. The definition of incident CVD/CEV was as follows: a hospitalization or a medical visit with a new diagnosis of coronary artery disease (CAD), myocardial infarction (MI), or stroke according to ICD-9 and ICD-10 from Med-Echo databases or RAMQ medical service files (ICD-9/ICD-10) (Table S11).

### 4.5. Covariates

We documented demographic variables upon cohort entry and determined the morbidity from the inpatient and outpatient ICD-9 and ICD-10 diagnostic codes recorded in the 3 years preceding the cohort entry (Table S11) [47,48,49]. We assessed all drug prescriptions filled in the year preceding the cohort entry (Table S12).

### 4.6. Statistical Analyses

Descriptive statistics were used to summarize the demographic and clinical characteristics of patients, according to adherent and non-adherent patient groups in the total cohort and sub-cohorts. The follow-up periods and adherence level were reported as the mean (standard deviation (SD)) and the median (first quartile (Q_1_) and third quartile (Q_3_)). Incident rates (per 100 person-years) of CVD and mortality were calculated for adherent and non-adherent groups in the total cohort and sub-cohorts.

For the main analyses, we used an IPTW approach to account for differences in patient characteristics between adherent and non-adherent groups [50,51]. We used a multivariable logistic regression model to estimate the observed probability (according to propensity score [PS] matching) of being in the adherent group (i.e., MPR ≥ 60%), based on all the baseline covariates, the length of the follow-up, and the year of cohort entry. By approximating the randomization used in randomized controlled trials, the IPTW approach establishes a pseudo-population, balances the treatment groups according to the covariates included in the model, and thus minimizes the impact of confounding biases in observational studies. All weights were stabilized by multiplying the IPTW weight by the marginal probability of being in the treatment group. Descriptive statistics were used to summarize the baseline characteristics of each IPTW group. For baseline characteristics, only absolute standardized differences of 10% or more between the unadjusted cohort and the IPTW-adjusted cohort were considered meaningful [51]. Hazard ratios with 95% CI were estimated using Cox proportional hazard models. Similar analyses were performed for sub-group cohorts, as well as for patients using typical and atypical antipsychotics for all cohorts.

For the sensitivity analyses of the CVD/CEV risk, we provided a negative control outcome analysis using the risk of glaucoma and hyperthyroidism in the total cohort. For the negative control, we assessed the risk of glaucoma, ICD-9: 365.x; ICD-10: H40, and hyperthyroidism, ICD-9: 240.x–242.x, 245.1; ICD-10: E05, E04.0–E04.2, E04.9, E01.2. We also calculated an E-value to assess the impact of unmeasured confounding for CVD/CEV and mortality risk [52]. The E-value indicates how strongly an unmeasured confounder would have to be associated with adherent versus non-adherent groups and the CVD/CEV and mortality to reduce the observed effect to the null, depending on the measured covariates. We explored the impact of doses used on mortality risk in total cohorts and sub-cohorts. The high and low doses were defined as being exposed to at least one claim of equivalent olanzapine of ≥10 mg and <10 mg after the index date, respectively [53,54]. We used an adjusted Cox model using (i) age, ≥75 vs. <75, and sex at the index date; (ii) 3 years prior the index date for associated morbidity, e.g., hypertension, diabetes, dyslipidemia, coronary artery disease (CAD, MI), heart failure, stroke, atrial fibrillation, major bleeding, chronic kidney disease, COPD, liver disease, neurologic diseases, and malign cancer; (iii) 1 year prior the index date for polypharmacy ≥ 10 vs. <10, hospitalizes, and users of antidepressants; and (iv) being users of prochlorperazine at index date (yes vs. no). All analyses were performed using SAS 9.4 statistical software (SAS Institute, Cary, NC, USA). A two-tailed *p*-value of less than 0.05 was considered statistically significant.

## 5. Conclusions

This study suggests that antipsychotic use in older adults does not seem to be linked with an increased CVD/CEV risk, except for atypical antipsychotic users in the sub-cohort of schizophrenia and for typical antipsychotic users in sub-cohort of dementia. The mortality risks were increased by 2- to 3-fold among typical antipsychotic users in all cohorts, except the sub-cohort of schizophrenia; these mortality risks were partly explained by being high dose users. Further studies are needed to clarify the effect of antipsychotic agents on stroke, CVD, and mortality risk in different populations, accounting for a dose selected and specific antipsychotic drug; in addition, they should rely on robust mental registries linking to other health data to facilitate control of confounders.

## Figures and Tables

**Table 1:** Demographic and clinical characteristics of the initial cohort and the cohort after IPTW according to the adherence level in the total cohort.

	Initial Cohort			Cohort after IPTW		
	Adherence Level			Adherence Level		
	<60% (n = 23,219)	≥60% (n = 19,431)	Absolute Standardized Difference	<60% (n = 23,219)	≥60% (n = 19,431)	Absolute Standardized Difference
Age at group entry, mean years (SD) *	78.7 (6.9)	81.7 (6.8)	0.43	80.4 (7.3)	80.4 (6.8)	0.01
Male, %	42.6	36.7	0.12	39.8	39.9	<0.01
**Prevalence within 3-year prior cohort entry, %**						
Hypertension	69.8	71.1	0.02	70.5	70.9	0.01
Diabetes mellitus	26.8	26.6	<0.01	26.8	27.2	0.01
Dyslipidemia	32.1	27.8	0.09	29.9	30.3	0.01
Stroke	12.9	16.0	0.09	14.9	15.1	0.01
Coronary artery disease excluding MI	44.4	45.0	0.01	45.1	45.8	0.01
Myocardial infarction	8.3	8.6	0.01	8.8	8.9	0.01
Heart failure	17.7	20.2	0.06	19.4	20.0	0.01
Atrial fibrillation	17.0	18.7	0.05	18.4	18.9	0.01
Major bleeding	12.9	12.0	0.03	12.5	12.8	0.01
Systemic embolism	1.7	1.3	0.03	1.6	1.7	0.01
CKD with eGRF < 30 mL/min ^†^	3.0	2.8	0.01	3.1	3.9	0.04
Acute renal failure	9.4	10.2	0.03	10.2	10.7	0.02
Liver disease	2.9	2.5	0.02	2.7	2.7	<0.01
COPD	38.6	36.9	0.04	38.1	38.5	0.01
Neurologic disease	20.9	26.6	0.14	24.6	24.9	0.01
Hypothyroidism	19.1	21.0	0.05	20.2	20.4	0.01
Malign cancer	52.9	28.8	0.51	40.6	39.6	0.02
Dementia	21.7	50.1	0.62	36.3	36.2	<0.01
**Medical procedures in 3-year prior cohort entry, %**						
Percutaneous coronary intervention/CABG	3.4	2.2	0.07	2.8	2.9	0.01
Medical procedures for a defibrillator	1.8	2.2	0.02	2.0	2.0	<0.01
**Medication in 1-year prior to cohort entry**						
Diuretics	35.5	37.4	0.04	36.9	37.4	0.01
Inhibitors of renin-angiotensin system	40.4	41.9	0.03	41.2	41.5	0.01
Beta-blockers	32.1	32.7	0.01	32.3	32.8	0.01
Spironolactone or eplerenone	3.0	2.9	0.01	3.0	3.1	<0.01
Digoxin	5.5	7.0	0.06	6.4	6.5	<0.01
Hydralazine	0.5	0.5	<0.01	0.5	0.5	<0.01
Nitrates	17.8	18.9	0.03	18.7	19.0	0.01
Statins	41.2	38.4	0.06	39.4	39.8	0.01
Antiarrhythmic (amiodarone or propafenone)	2.4	2.3	0.01	2.4	2.4	<0.01
Warfarin	12.0	12.4	0.01	12.4	12.8	0.01
DOAC	0.2	0.1	0.01	0.1	0.1	<0.01
Antiplatelets (without ASA)	7.2	8.1	0.04	8.0	8.0	<0.01
Low-dose ASA	44.0	47.1	0.06	45.7	46.3	0.01
Antidiabetics						
Metformin	13.1	12.9	0.01	12.9	13.0	<0.01
Sulfonylurea	8.9	8.9	<0.01	8.9	9.1	0.01
Thiazolidinediones	1.7	1.7	0.01	1.7	1.7	<0.01
DPP-4 inhibitors	0.2	0.2	<0.01	0.2	0.2	<0.01
SGLT2 inhibitors	0.0	0.0	-	0.0	0.0	-
Insulins	3.7	3.7	<0.01	3.8	4.1	0.01
**Other medications**						
Proton pump inhibitors	42.4	37.9	0.09	40.6	41.0	0.01
Antidepressant agents	29.1	38.7	0.21	34.5	35.0	0.01
Anticholinergics agents	4.5	3.7	0.04	4.2	4.3	0.01
Benzodiazepine	49.6	52.6	0.06	51.4	52.2	0.02
Polypharmacy (≥10 medications)	56.5	54.0	0.05	55.9	56.8	0.02
**Health care services in 1-year prior cohort entry**						
Number of visit medicals, mean (SD) *	11.5 (11.2)	8.4 (9.7)	0.30	10.0 (10.0)	11.0 (17.6)	0.07
Emergency visit, mean (SD) *	2.0 (2.7)	2.1 (3.0)	0.02	2.1 (2.9)	2.2 (2.9)	0.01
Hospitalization (%)	60.8	53.7	0.14	57.6	57.4	<0.01

*: Mean ± SD (standard deviation); ^†^: CKD: Chronic renal failure with estimated Glomerular rate filtration < 30 mL/min. Abbreviations: COPD: chronic obstructive pulmonary disease; ASA: acid acetylic salicylic acid; CABG: coronary artery bypass graft surgery; MI: myocardial infarction; DOAC: direct oral anticoagulants; DPP-4 inhibitors: dipeptidyl peptidase; IPTW: inverse probability of treatment weighting; SGLT2 inhibitors: sodium-glucose co-transporter 2.

**Table 2:** Hazard ratios (95% CI) for CVD/CEV outcomes and mortality in the total cohort and sub-cohorts according to the adherence ≥ 60% vs. <60% (reference) after IPTW-marching according to the adherence level among total antipsychotics, typical antipsychotics, and atypical antipsychotics.

		CVD/CEV Outcomes		Mortality	
		HR (95% CI)	*p*-Value	HR (95% CI)	*p*-Value
**Total cohort**	Adherence ≥ 60% (ref.: <60%) of total antipsychotics	0.92 (0.89–0.96)	<0.0001	0.96 (0.94–0.98)	0.0005
	Adherence ≥ 60% (ref.: <60%) of atypical antipsychotics	0.93 (0.90–0.96)	<0.0001	0.74 (0.72–0.75)	<0.0001
	Adherence ≥ 60% (ref.: <60%) of typical antipsychotics	0.96 (0.87–1.06)	0.3680	3.35 (3.22–3.50)	<0.0001
**Sub-cohort without schizophrenia/dementia**	Adherence ≥ 60% (ref.: <60%) of total antipsychotics	0.94 (0.90–0.99)	0.0086	0.98 (0.94–1.01)	0.1272
	Adherence ≥ 60% (ref.: <60%) of atypical antipsychotics	0.97 (0.92–1.01)	0.1239	0.67 (0.65–0.70)	<0.0001
	Adherence ≥ 60% (ref.: <60%) of typical antipsychotics	0.79 (0.69–0.91)	0.0007	3.58 (3.40–3.76)	<0.0001
**Sub-cohort of patients with schizophrenia**	Adherence ≥ 60% (ref.: <60%) of total antipsychotics	1.37 (1.06–1.77)	0.0165	0.92 (0.74–1.15)	0.4698
	Adherence ≥ 60% (ref.: <60%) of atypical antipsychotics	1.33 (1.04–1.70)	0.0233	0.88 (0.71–1.09)	0.2320
	Adherence ≥ 60% (ref.: <60%) of typical antipsychotics	0.89 (0.49–1.62)	0.7053	1.25 (0.77–2.02)	0.3695
**Sub-cohort of patients with dementia**	Adherence ≥ 60% (ref.: <60%) of total antipsychotics	0.89 (0.83–0.94)	<0.0001	0.98 (0.94–1.02)	0.3141
	Adherence ≥ 60% (ref.: <60%) of atypical antipsychotics	0.87 (0.84–0.91)	<0.0001	0.88 (0.84–0.91)	<0.0001
	Adherence ≥ 60% (ref.: <60%) of typical antipsychotics	1.25 (1.04–1.51)	0.0153	2.45 (2.23–2.69)	<0.0001

Abbreviations: CI: confidence interval; HR: hazard ratios.

**Table 3:** Hazard ratios (95% CI) of exploring the dose used on the **mortality** risk in the total cohort and the sub-cohorts among users of typical antipsychotic agents.

		HR (95% CI)	*p*-Value
**Total cohort**	Users of high dose vs. low dose of typical antipsychotic agents (≥10 mg vs. <10 mg Eq-Olan) *	1.67 (1.59–1.74)	<0.0001
**Sub-cohort without schizophrenia/dementia**	Users of high dose vs. low dose of typical antipsychotic agents (≥10 mg vs. <10 mg Eq-Olan)	1.64 (1.56–1.73)	<0.0001
**Sub-cohort of patients with schizophrenia**	Users of high dose vs. low dose of typical antipsychotic agents (≥10 mg vs. <10 mg Eq-Olan)	1.24 (0.74–2.09)	0.4142
**Sub-cohort of patients with dementia**	Users of high dose vs. low dose of typical antipsychotic agents (≥10 mg vs. <10 mg Eq-Olan)	1.69 (1.53–1.88)	<0.0001

* Eq-Olan: Equivalent olanzapine. Abbreviations: CI: confidence interval; HR: hazard ratios.

**Table 4:** Sensitivity analysis of negative controls after IPTW according to the adherence level in the total cohort for the cardiovascular events.

	Incident Rate of ≥60% 100 PY (95% CI)	Incident Rate of <60 100 PY (95% CI)	HR (95% CI)	*p*-Value
Glaucoma	0.4 (0.3–0.6)	0.3 (0.2–0.5)	1.25 (0.71–2.22)	0.4410
Hyperthyroidism	0.08 (0.01–0.16)	0.11 (0.03–0.20)	0.72 (0.23–2.27)	0.5754

Abbreviations: CI: confidence interval; HR: hazard ratios; PY, person-years.

**Table 5:** E-values for significant comparison analysis in the total cohort and the sub-cohorts after IPTW of ≥60% vs. <60% of the CDV/CEV and mortality risks.

			HR (95% CI)	E-Value Corresponding to the CI Bound Closest to 1	E-Value for HR Point Estimate
**Total cohort**	CVD/CEV risk	Atypical antipsychotics	0.93 (0.90–0.96)	1.25	1.36
	Mortality risk	Typical antipsychotics	3.55 (3.39–3.71)	6.24	6.56
		Atypical antipsychotics	0.67 (0.65–0.69)	2.26	2.35
**Sub-cohort without schizophrenia/dementia**	CVD/CEV risk	Typical antipsychotics	0.79 (0.69–0.91)	1.43	1.85
	Mortality risk	Typical antipsychotics	3.58 (3.40–3.76)	6.26	6.62
		Atypical antipsychotics	0.67 (0.65–0.70)	2.21	2.35
**Sub-cohort with schizophrenia**	CVD/CEV risk	Atypical antipsychotics	1.33 (1.04–1.70)	1.24	1.99
**Sub-cohort with dementia**	CVD/CEV risk	Typical antipsychotics	1.25 (1.04–1.51)	1.24	1.81
		Atypical antipsychotics	0.87 (0.82–0.92)	1.39	1.56
	Mortality risk	Typical antipsychotics	2.45 (2.23–2.69)	3.89	4.33
		Atypical antipsychotics	0.88 (0.84–0.71)	1.67	1.53

Abbreviations: CI: confidence interval; CVD: cardiovascular disease; HR: hazard ratios; PY, person-years.

## Data Availability

Data access requests for the RAMQ databases are approved by the *Commission d’accès à l’information du Québec*. All data generated or analyzed during this study are included in this published article and its supplementary information file.

## Supplementary Materials

The following supporting information can be downloaded at: https://www.mdpi.com/article/10.3390/ph17020178/s1.
