# Supplementary material for: Antipsychotics and Risks of Cardiovascular and Cerebrovascular Diseases and Mortality in Dwelling Community Older Adults
Source: Pharmaceuticals (Basel). 2024 Jan 30;17(2):178. doi: 10.3390/ph17020178 (PMC10892683; doi:10.3390/ph17020178)
Supplement: Supplementary file 1 [file pharmaceuticals-17-00178-s001.zip › pharmaceuticals-2821113-supplementary.pdf]

Supplemental files

Table S1 Demographic and clinical characteristics of the initial sub-cohort without schizophrenia/dementia and after IPTW according to the adherence level.

	**Initial sub-cohort**			**Sub-cohort after IPTW**		
	Adherence level			Adherence level		
	< 60% (n=18,002)	≥ 60% (n=9,398)	Absolute standardized difference	< 60% (n=18,002)	≥ 60% (n=9,398)	Absolute standardized difference
Age at group entry, mean years (SD)*	77.6 (6.5)	80.7 (7.2)	0.47	78.8 (7.0)	79.0 (6.8)	0.03
Male, %	44.0	39.0	0.10	42.2	42.5	0.01
**Prevalence within 3-year prior cohort entry, %**						
Hypertension	68.9	69.6	0.01	69.3	69.9	0.01
Diabetes mellitus	26.8	27.1	0.01	27.1	27.9	0.02
Dyslipidemia	32.9	28.4	0.10	31.3	31.6	0.01
Stroke	11.0	14.0	0.09	12.3	13.0	0.02
Coronary artery disease excluding MI	43.3	45.1	0.04	44.3	45.7	0.03
Myocardial infarction	7.8	8.7	0.03	8.2	8.7	0.02
Heart failure	16.3	20.5	0.11	18.2	19.3	0.03
Atrial fibrillation	15.6	18.5	0.08	16.9	18.0	0.03
Major bleeding	12.8	11.7	0.03	12.4	12.8	0.01
Systemic embolism	1.7	1.5	0.01	1.7	2.0	0.02
CKD with eGRF < 30 mL/min [†]	2.9	3.2	0.01	3.1	4.4	0.07
Acute renal failure	8.4	9.6	0.04	9.1	10.0	0.03
Liver disease	2.8	2.7	0.01	2.7	2.8	<0.01
COPD[‡]	38.9	39.5	0.01	39.3	40.1	0.02
Neurologic disease	16.7	21.8	0.13	18.8	19.7	0.02
Hypothyroidism	18.3	19.3	0.03	18.9	19.3	0.01
Malign cancer	59.9	34.9	0.52	50.7	48.7	0.04
Dementia	0.0	0.0	-	0.0	0.0	-
**Medical procedures in 3-year prior to cohort entry, %**						
Percutaneous coronary intervention /CABG	3.7	2.9	0.04	3.4	3.5	0.01
Medical procedures for a defibrillator	1.7	2.0	0.02	1.9	2.0	0.01
**Medication in 1-year prior to cohort entry**						
Diuretics	34.9	39.2	0.09	36.7	37.9	0.03
Inhibitors of renin-angiotensin system	40.4	42.7	0.05	41.4	41.8	0.01
Beta-blockers	32.4	34.2	0.04	33.2	34.0	0.02
Spironolactone or eplerenone	3.0	3.3	0.02	3.2	3.2	<0.01
Digoxin	5.0	7.0	0.09	5.8	6.0	0.01
Hydralazine	0.4	0.6	0.02	0.5	0.5	<0.01
Nitrates	17.5	20.9	0.08	18.9	19.5	0.01

Statins	43.1	40.4	0.01	42.1	42.5	0.01
Antiarrhythmic (amiodarone or propafenone)	2.5	2.6	0.01	2.5	2.5	<0.01
Warfarin	11.6	12.9	0.04	12.2	12.8	0.02
DOAC	0.2	0.1	<0.01	0.2	0.1	<0.01
Antiplatelets (without ASA)[¥]	6.5	8.1	0.06	7.1	7.4	0.01
Low-dose ASA	42.8	46.3	0.07	44.2	45.3	0.02
**Antidiabetics**						
Metformin	13.3	13.5	0.01	13.5	13.5	<0.01
Sulfonylurea	9.0	9.1	<0.01	9.1	9.4	0.01
Thiazolidinediones	1.8	1.9	<0.01	1.8	1.9	0.01
DPP-4 inhibititors[£]	0.2	0.2	<0.01	0.2	0.3	0.01
SGLT2 inhibitors[£]	0.0	0.0	-	0.0	0.0	-
Insulins	3.6	4.1	0.03	3.9	4.2	0.02
**Other medications**						
Proton pump inhibitors	43.7	42.0	0.03	43.4	44.4	0.02
Antidepressant agents	27.8	40.6	0.27	32.9	34.4	0.03
Anticholinergics agents	4.8	4.7	<0.01	4.8	4.8	<0.01
Benzodiazepine	51.5	59.3	0.16	54.6	56.2	0.03
Polypharmacy ($\geq$ 10 medications)	57.7	59.5	0.04	58.9	60.6	0.04
**Health care services in 1-year prior**						
Number of visit medicals, mean (SD)	12.4 (11.5)	9.2 (10.6)	0.29	11.3 (10.6)	12.6 (19.7)	0.08
Emergency visit, mean (SD)	1.9 (2.6)	2.0 (2.8)	0.05	2.0 (2.7)	2.0 (2.7)	0.02
Hospitalization (%)	60.0	49.7	0.21	56.5	56.1	0.01

[*]: Mean ±SD (Standard Deviation)

[†]: CKD: Chronic renal failure with estimated Glomerular rate filtration < 30 mL/min.

[‡]: COPD: Chronic obstructive pulmonary disease

[¥]: ASA: Acid acetylic salicylic acid

[£]: DPP-4 inhibitors: Dipeptidyl peptidase; SGLT2 inhibitors: Sodium-glucose co-transporter 2 inhibitors.

MI: Myocardial infarction.

Table S2. Demographic and clinical characteristics of the initial sub-cohort of schizophrenia and after IPTW according to the adherence level.

	Initial sub-cohort			Sub-cohort after IPTW		
	Adherence level			Adherence level		
	< 60% (n=278)	≥ 60% (n=513)	Absolute standardized difference	< 60% (n=278)	≥ 60% (n=513)	Absolute standardized difference
Age at group entry, mean years (SD)*	77.3 (6.6)	78.4 (7.0)	0.17	78.0 (6.7)	78.0 (7.0)	<0.01
Male, %	33.5	30.4	0.07	30.7	31.6	0.02
**Prevalence within 3-year prior cohort entry, %**						
Hypertension	61.9	63.4	0.03	62.5	63.0	0.01
Diabetes mellitus	18.7	21.3	0.06	18.4	19.8	0.04
Dyslipidemia	22.7	24.4	0.04	22.7	24.0	0.03
Stroke	11.9	13.5	0.05	14.0	13.2	0.02
Coronary artery disease excluding MI	35.6	34.5	0.02	34.2	34.8	0.01
Myocardial infarction	6.5	5.3	0.05	5.3	5.5	0.01
Heart failure	13.0	14.4	0.04	14.4	13.7	0.02
Atrial fibrillation	13.3	13.1	0.01	13.4	13.2	0.01
Major bleeding	12.2	9.6	0.09	11.0	10.8	0.01
Systemic embolism	0.4	1.2	0.09	0.8	0.9	0.01
CKD with eGRF < 30 mL/min †	2.2	2.5	0.02	2.3	2.4	0.01
Acute renal failure	6.8	6.0	0.03	6.0	6.1	<0.01
Liver disease	1.8	2.1	0.02	2.0	1.9	0.01
COPD‡	36.7	28.9	0.17	31.8	31.7	<0.01
Neurologic disease	24.1	21.1	0.07	22.3	22.5	<0.01
Hypothyroidism	19.4	23.2	0.09	20.9	21.9	0.03
Malign cancer	22.3	20.7	0.04	21.2	21.2	<0.01
Dementia	34.5	40.9	0.13	38.2	38.5	0.01
**Medical procedures in 3-year prior to cohort entry, %**						
Percutaneous coronary intervention /CABG	2.2	1.6	0.04	1.5	1.6	0.01
Medical procedures for a defibrillator	0.7	1.4	0.06	1.4	1.2	0.02
**Medications in 1-year prior to cohort entry**						
Diuretics	27.0	27.1	<0.01	28.3	27.0	0.03
Inhibitors of renin-angiotensin system	28.1	32.0	0.08	30.2	30.5	0.01
Beta-blockers	21.9	27.5	0.13	24.4	25.4	0.02
Spironolactone or eplerenone	1.8	1.0	0.07	1.1	1.1	<0.01
Digoxin	3.2	3.1	0.01	3.3	3.1	0.01
Hydralazine	0.4	0.2	0.03	0.2	0.2	<0.01
Nitrates	12.2	12.3	<0.01	11.7	12.1	0.01
Statins	22.3	29.6	0.17	24.8	26.9	0.05

Antiarrhythmic (amiodarone or propafenone)	2.2	1.4	0.06	1.5	1.6	<0.01
Warfarin	8.3	7.6	0.02	8.4	7.9	0.02
DOAC	0.0	0.2	0.06	0.0	0.1	0.05
Antiplatelets (without ASA)[¥]	5.8	4.1	0.08	4.4	4.6	0.01
Low-dose ASA	32.4	37.8	0.11	34.0	35.3	0.03
**Antidiabetics**						
Metformin	6.8	10.3	0.13	8.0	8.9	0.03
Sulfonylurea	5.4	7.4	0.08	6.3	6.6	0.01
Thiazolidinediones	1.1	1.6	0.04	1.4	1.4	<0.01
DPP-4 inhibititors[£]	0.0	0.4	0.09	0.0	0.3	0.07
SGLT2 inhibitors[£]	0.0	0.0	-	0.0	0.0	-
Insulins	2.2	2.7	0.04	2.2	2.5	0.02
**Other medications**						
Proton pump inhibitors	28.1	26.3	0.04	27.0	26.7	0.01
Antidepressant agents	36.7	38.6	0.04	38.6	38.4	<0.01
Anticholinergics agents	4.0	3.3	0.03	3.3	3.4	<0.01
Benzodiazepine	48.9	51.5	0.05	50.0	50.3	0.01
Polypharmacy (≥ 10 medications)	40.3	39.6	0.01	38.6	39.4	0.02
**Health care services in 1-year prior**						
Number of visit medicals, mean (SD)	9.2 (9.5)	8.3 (11.9)	0.08	8.6 (8.5)	8.7 (13.0)	0.01
Emergency visit, mean (SD)	2.7 (3.3)	2.8 (7.7)	0.03	2.7 (3.3)	2.8 (7.0)	0.01
Hospitalization (%)	59.4	57.1	0.05	57.3	57.8	0.01

*: Mean ±SD (Standard Deviation)

†: CKD: Chronic renal failure with estimated Glomerular rate filtration < 30 mL/min.

‡: COPD: Chronic obstructive pulmonary disease

¥: ASA: Acid acetylic salicylic acid

£: DPP-4 inhibitors: Dipeptidyl peptidase; SGLT2 inhibitors: Sodium-glucose co-transporter 2 inhibitors.

Table S3. Demographic and clinical characteristics of the initial sub-cohort of dementia and after IPTW according to the adherence level.

	**Initial sub-cohort**			**Sub-cohort after IPTW**		
	Adherence level			Adherence level		
	< 60% (n=5,035)	≥ 60% (n=9,730)	Absolute standardized difference	< 60% (n=5,035)	≥ 60% (n=9,730)	Absolute standardized difference
Age at group entry, mean years (SD)*	82.8 (6.5)	82.7 (6.3)	0.01	82.8 (6.5)	82.7 (6.3)	<0.01
Male, %	38.0	34.7	0.07	36.1	35.9	<0.01
**Prevalence within 3-year prior cohort entry, %**						
Hypertension	73.3	72.9	0.01	72.8	73.0	<0.01
Diabetes mellitus	26.8	26.2	0.01	26.5	26.5	<0.01
Dyslipidemia	29.7	27.5	0.05	28.0	28.2	<0.01
Stroke	19.9	18.2	0.04	18.8	18.8	<0.01
Coronary artery disease excluding MI	48.4	45.3	0.06	46.4	46.4	<0.01
Myocardial infarction	10.4	8.5	0.06	9.3	9.2	<0.01
Heart failure	22.7	22.1	0.06	21.1	21.0	<0.01
Atrial fibrillation	22.1	19.2	0.07	20.3	20.2	<0.01
Major bleeding	13.7	12.3	0.04	12.7	12.8	<0.01
Systemic embolism	1.7	1.1	0.04	1.3	1.3	<0.01
CKD with eGRF < 30 mL/min †	3.3	2.5	0.05	2.8	2.8	<0.01
Acute renal failure	13.1	10.9	0.07	11.7	11.7	<0.01
Liver disease	3.0	2.4	0.04	2.6	2.6	<0.01
COPD‡	37.6	34.6	0.06	35.7	35.7	<0.01
Neurologic disease	36.0	31.5	0.09	33.1	33.1	<0.01
Hypothyroidism	22.0	22.5	0.01	22.4	22.4	<0.01
Malign cancer	29.3	23.2	0.14	25.3	25.3	<0.01
**Medical procedures in 3-year prior to cohort entry, %**						
Percutaneous coronary intervention /CABG	2.1	1.5	0.05	1.7	1.7	<0.01
Medical procedures for a defibrillator	2.2	2.4	0.01	2.3	2.3	<0.01
**Medications in 1-year prior to cohort entry**						
Diuretics	38.3	36.0	0.04	36.8	36.8	<0.01
Inhibitors of renin-angiotensin system	40.8	41.4	0.01	41.2	41.2	<0.01
Beta-blockers	31.3	31.3	<0.01	31.4	31.3	<0.01
Spironolactone or eplerenone	3.2	2.6	0.04	2.8	2.8	<0.01
Digoxin	7.4	7.1	0.01	7.2	7.2	<0.01
Hydralazine	0.6	0.4	0.03	0.5	0.5	<0.01
Nitrates	19.2	17.2	0.05	17.9	17.9	<0.01
Statins	35.2	36.8	0.03	36.0	36.2	<0.01
Antiarrhythmic (amiodarone or propafenone)	2.4	2.0	0.03	2.1	2.1	<0.01

Warfarin	13.6	12.1	0.05	12.7	12.6	<0.01
DOAC	0.1	0.1	0.03	0.1	0.1	<0.01
Antiplatelets (without ASA)[¥]	9.6	8.3	0.05	8.9	8.8	<0.01
Low-dose ASA	48.4	48.2	<0.01	48.1	48.3	<0.01
**Antidiabetics**						
Metformin	12.3	12.3	<0.01	12.2	12.3	<0.01
Sulfonylurea	8.7	8.6	<0.01	8.7	8.7	<0.01
Thiazolidinediones	1.5	1.5	<0.01	1.5	1.5	<0.01
DPP-4 inhibititors[£]	0.1	0.2	0.01	0.1	0.1	<0.01
SGLT2 inhibitors[£]	0.0	0.0	-	0.0	0.0	-
Insulins	4.1	3.3	0.04	3.6	3.6	<0.01
**Other medications**						
Proton pump inhibitors	38.3	34.3	0.08	35.7	35.7	<0.01
Antidepressant agents	33.3	36.9	0.07	35.6	35.7	<0.01
Anticholinergics agents	3.7	2.7	0.05	3.0	3.0	<0.01
Benzodiazepine	43.1	46.2	0.06	45.1	45.1	<0.01
Polypharmacy (≥10 medications)	52.7	49.1	0.07	50.4	50.4	<0.01
**Health care services in 1-year prior**						
Number of visit medicals, mean (SD)	8.5 (9.9)	7.6 (8.7)	0.10	8.0 (8.7)	8.0 (10.0)	<0.01
Emergency visit, mean (SD)	2.5 (3.1)	2.2 (3.2)	0.10	2.3 (2.9)	2.3 (4.0)	<0.01
Hospitalization (%)	63.8	57.7	0.12	59.8	59.8	<0.01

[*]: Mean ±SD (Standard Deviation)

[†]: CKD: Chronic renal failure with estimated Glomerular rate filtration < 30 mL/min.

[‡]: COPD: Chronic obstructive pulmonary disease

[¥]: ASA: Acid acetylic salicylic acid

[£]: DPP-4 inhibitors: Dipeptidyl peptidase; SGLT2 inhibitors: Sodium-glucose co-transporter 2 inhibitors.

Table S4. Frequency of use, olanzapine equivalent and users ≥ 10 mg in total cohort and sub-cohorts.

**Atypical antipsychotics**	**Profile of use at initiation in total cohort, N (%)**	**Equivalent olanzapine in total cohort Mean (SD)/Median at initiation and at 1 year (mg)**	**% ≥10 mg olanzapine equivalent at initiation and at 1 year in total cohort**
Aripiprazole	98 (0.2%)	Initiation:1.7 (1.3) / 1.3<br>1 year: 1.8 (1.3)/ 1.3	Initiation:1.0%<br>1 year:0.0%
Olanzapine	4,076 (9.6%)	Initiation:3.4 (1.8) / 2.5<br>1 year:3.4 (3.5) / 2.5	Initiation:3.3%<br>1 year:4.3%
Quetiapine	9,089 (21.3%)	Initiation:0.7 (0.4) / 0.6<br>1 year:1.3 (9.9) / 0.6	Initiation:<0.1%<br>1 year:0.5%
Risperidone	14,127 (33.1%)	Initiation:0.8 (1.0) / 0.5<br>1 year:1.1 (3.0) / 0.6	Initiation:<0.1%<br>1 year:0.6%
Other atypical antipsychotic agents*	85 (< 0.2%)	Initiation:1.1 (1.4) / 0.5<br>1 year:1.1 (1.1) / 0.6	Initiation:1.2%<br>1 year:0.0%
**Typical antipsychotic**	15,175 (35.6%)	Initiation:10.3 (54.5) / 1.0<br>1 year: 39.1 (109.1) / 1.0	Initiation: 5.1%<br>1 year: 21.4%
Haloperidol	3,529 (8.3%)	Initiation:2.0 (5.3) / 0.6<br>1 year: 1.8 (8.2) / 0.6	Initiation:0.4%<br>1 year:1.0%
Prochlorperazine	10,128 (23.7%)	Initiation:0.9 (0.2) / 1.0<br>1 year: 4.8 (34.4) / 1.0	Initiation:0.0%<br>1 year:2.5%
Methotrimeprazine	733 (1.7%)	Initiation:188.7 (167.0)/74.6<br>1 year: 168.6 (177.2) / 74.6	Initiation:100%<br>1 year:91.6%
**Atypical antipsychotics**	**Profile of use at initiation in sub-cohort without schizophrenia/dementia, N (%)**	**Equivalent olanzapine (mg) in sub-cohort Mean (SD)/Median at initiation and at 1 year**	**% ≥10 mg olanzapine equivalent at initiation and at 1 year in sub-cohort**
Aripiprazole	74 (0.3%)	Initiation:1.7 (1.4) / 1.3<br>1 year: 1.7 (1.4)/ 1.3	Initiation:1.4%<br>1 year:0.0%
Olanzapine	2,365 (8.6%)	Initiation:3.4 (1.8) / 2.5<br>1 year:3.4 (3.2) / 2.5	Initiation:3.6%<br>1 year:4.5%
Quetiapine	5,552 (20.3%)	Initiation:0.7 (0.5) / 0.6<br>1 year:1.4 (10.8) / 0.6	Initiation:<0.1%<br>1 year:0.7%
Risperidone	6,125 (22.4%)	Initiation:0.8 (1.1) / 0.5<br>1 year:1.1 (2.8) / 0.6	Initiation:<0.1%<br>1 year:0.6%
Other atypical antipsychotic agents*	42 (< 0.2%)	Initiation:1.1 (1.0) / 0.5<br>1 year:1.4 (1.5) / 0.8	Initiation:0.0%<br>1 year:0.0%
**Typical antipsychotic**	13,242 (48.3%)	Initiation:10.2 (54.6) / 1.0<br>1 year: 49.7 (122.9) / 1.0	Initiation: 5.0%<br>1 year: 26.1%
Haloperidol	2,290 (8.4%)	Initiation:2.0 (4.5) / 0.6<br>1 year:1.7 (8.0) / 0.6	Initiation:0.2%<br>1 year:0.4%
Prochlorperazine	9,607 (35.1%)	Initiation:0.9 (0.2) / 1.0<br>1 year:5.2 (36.1) / 1.0	Initiation:0.0%<br>1 year:2.5%
Methotrimeprazine	648 (2.4%)	Initiation:185.4 (168.8)/74.6<br>1 year: 175.1 (180.6) / 74.6	Initiation:100%<br>1 year:92.9%
**Atypical antipsychotics**	**Profile of use at initiation in sub-cohort of schizophrenia, N (%)**	**Equivalent olanzapine in cohort of schizophrenia Mean (SD)/Median (mg)**	**% ≥10 mg olanzapine equivalent in cohort of schizophrenia**
Aripiprazole	2 (0.3%)	Initiation:2.3 (1.4) / 2.3<br>1 year: -	Initiation:0.0%<br>1 year:0.0%

Olanzapine	177 (22.4%)	Initiation:4.6 (2.6) / 5.0<br>1 year:5.1 (8.9) / 2.5	Initiation:11.3%<br>1 year:12.6%
Quetiapine	148 (18.7%)	Initiation:1.0 (1.1) / 1.6<br>1 year:2.0 (2.1) / 0.6	Initiation:0.0%<br>1 year:1.1%
Risperidone	364 (46.0%)	Initiation:1.2 (1.1) / 1.0<br>1 year:2.6 (6.0) / 1.0	Initiation:0.0%<br>1 year:3.7%
Other atypical antipsychotic agents*	7 (0.9%)	ND	ND
**Typical antipsychotics**	93 (11.8%)	Initiation: 33.9 (86.7) / 1.3<br>1 year: 25.3 (63.2) / 2.0	Initiation: 24.7%<br>1 year: 26.8%
Haloperidol	48 (6.1%)	Initiation:8.1 (22.9) / 1.3<br>1 year:9.9 (28.8) / 1.1	Initiation:10.4%<br>1 year:9.1%
Prochlorperazine	17 (2.1%)	Initiation:1.0 (0.1) / 1.0<br>1 year:10.0 (n=1)	Initiation:0.0%<br>1 year:100.0% (n=1)
Methotrimeprazine	7 (1.9%)	Initiation: 287.8 (145.7)/ 373.1<br>1 year: 99.7 (138.8) / 74.6	Initiation:100%<br>1 year:66.7%
**Atypical antipsychotics**	**Profile of use at initiation in sub-cohort of dementia, N (%)**	**Equivalent olanzapine in cohort of dementia<br>Mean (SD)/Median (mg)**	**% ≥10 mg olanzapine equivalent in cohort of dementia**
Aripriprazole	22 (0.2%)	Initiation:1.8 (1.2) / 1.3<br>1 year: 1.9 (1.3)/ 1.3	Initiation:0.0%<br>1 year:0.0%
Olanzapine	1,598 (10.8%)	Initiation:3.4 (1.6) / 2.5<br>1 year:3.2 (3.8) / 2.5	Initiation:2.2%<br>1 year:3.2%
Quetiapine	3,441 (23.3%)	Initiation:0.7 (0.4) / 0.6<br>1 year:1.1 (8.8) / 0.6	Initiation:0.0%<br>1 year:0.2%
Risperidone	7,797 (52.8%)	Initiation:0.8 (1.0) / 0.5<br>1 year:1.1 (2.9) / 0.5	Initiation:<0.1%<br>1 year:0.4%
Other atypical antipsychotic agents	39 (0.3%)	Initiation:0.7 (0.4) / 0.5<br>1 year:0.8 (0.5) / 0.6	Initiation:<0.1%<br>1 year:0.0%
**Typical antipsychotic**	1,868 (12.7%)	Initiation:10.4 (51.7) / 1.0<br>1 year: 7.7 (37.6) / 0.6	Initiation: 4.8%<br>1 year: 6.1%
Haloperidol	1,211 (8.2%)	Initiation:1.9 (6.1) / 0.5<br>1 year:1.6 (7.7) / 0.6	Initiation:0.5%<br>1 year:1.1%
Prochlorperazine	506 (3.4%)	Initiation:0.9 (0.2) / 1.0<br>1 year:1.0 (1.0) / 0.6	Initiation:0.0%<br>1 year:0.0%
Methotrimeprazine	78 (0.5%)	Initiation: 207.4 (150.6)/ 74.6<br>1 year: 101.2 (117.5) / 74.6	Initiation:100%<br>1 year:81.0%

*Other atypical antipsychotic agents: clozapine, loxapine, lurasidone and ziprasidone.

Table S5. Proportion of the level of adherence in the 5-year period of follow-up in the total cohort

cat_adhesion_antipsych_5yrs_fup	Frequency	Percent	Cumulative Frequency	Cumulative Percent
1) ADHÉSION < 50%	21509	50.43	21509	50.43
2) ADHÉSION 50%-59%	1710	4.01	23219	54.44
3) ADHÉSION 60%-69%	1834	4.30	25053	58.74
4) ADHÉSION 70%-79%	2056	4.82	27109	63.56
5) ADHÉSION 80%-89%	2981	6.99	30090	70.55
6) ADHÉSION 90%-99%	6576	15.42	36666	85.97
7) ADHÉSION 100%	5984	14.03	42650	100.00

Table S6. Hazard ratios (95% CI) for CVD/CEV and mortality risks according to the adherence ≥ 60% vs < 60% (reference) after IPTW matching in the total cohort and sub-cohorts for all antipsychotic users.

**Total cohort**	Incident rates in the 5-year period, per 100 person-years (95% CI) after IPTW matching		Hazard ratios (95% CI) (ref.: < 60%)	p-value
	< 60% (n=23,219)	≥ 60% (n=19,431)		
CVD/CEV events				
CAD	9.6 (9.4-9.9)	8.6 (8.3-8.8)	0.89 (0.86-0.93)	<0.0001
Stroke	4.9 (4.7-5.1)	4.6 (4.4-4.8)	0.95 (0.90-0.99)	0.0383
MI	1.7 (1.6-1.8)	1.6 (1.5-1.7)	0.96 (0.88-1.05)	0.3317
All CVD/CEV	14.4 (14.1-14.8)	13.3 (12.9-13.6)	0.92 (0.89-0.96)	<0.0001
All-cause mortality	22.1 (21.8-22.5)	21.0 (20.6-21.4)	0.96 (0.94-0.98)	0.0005

**Sub-cohort without schizophrenia/dementia**	Incident rates in the 5-year period, per 100 person-years (95% CI) after IPTW matching		Hazard ratios (95% CI) (ref.: < 60%)	p-value
	< 60% (n=18,002)	≥ 60% (n=9,398)		
CVD/CEV events				
CAD	10.4 (10.1-10.7)	9.4 (9.1-9.8)	0.90 (0.86-0.95)	<0.0001
Stroke	4.5 (4.3-4.7)	4.3 (4.1-4.6)	0.96 (0.89-1.03)	0.2302
MI	1.8 (1.6-1.9)	1.7 (1.5-1.8)	0.96 (0.86-1.08)	0.5147
All CVD/CEV	14.7 (14.3-15.0)	13.8 (13.3-14.3)	0.94 (0.90-0.99)	0.0086
All-cause mortality	21.1 (20.7-21.5)	20.1 (19.6-20.7)	0.98 (0.94-1.01)	0.1272

**Sub-cohort of patients with schizophrenia**	Incident rates in the 5-year period, per 100 person-years (95% CI) after IPTW matching		Hazard ratios (95% CI) (ref.: < 60%)	p-value
	< 60% (n=278)	≥ 60%(n=513)		
CVD/CEV events				
CAD	5.8 (4.3-7.4)	7.4 (6.1-8.7)	1.27 (0.93-1.74)	0.1320
Stroke	3.7 (2.5-4.9)	4.7 (3.7-5.7)	1.28 (0.87-1.88)	0.2085
MI	1.1 (0.4-1.7)	1.5 (0.9-2.0)	1.39 (0.70-2.77)	0.3487
All CVD/CEV	8.8 (6.9-10.7)	12.1 (10.4-13.8)	1.37 (1.06-1.77)	0.0165
All-cause mortality	11.5 (9.5-13.6)	10.6 (9.2-12.1)	0.92 (0.74-1.15)	0.4698

**Sub-cohort of patients with dementia**	Incident rates in the 5-year period, per 100 person-years (95% CI) after IPTW matching		Hazard ratios (95% CI) (ref.: < 60%)	p-value
	< 60% (n=9,750)	≥ 60% (n=5,035)		
CVD/CEV events				
CAD	8.1 (7.6-8.6)	7.2 (6.9-7.5)	0.89 (0.83-0.96)	0.0022
Stroke	5.5 (5.1-5.9)	4.9 (4.6-5.1)	0.88 (0.81-0.96)	0.0059
MI	1.6 (1.4-1.8)	1.5 (1.3-1.6)	0.91 (0.78-1.07)	0.2422
All CVD/CEV	13.9 (13.3-14.6)	12.3 (11.8-12.7)	0.89 (0.83-0.94)	<0.001
All-cause mortality	23.4 (22.6-24.2)	22.9 (22.3-23.4)	0.98 (0.94-1.02)	0.3141

CVD/CEV: Cardiovascular and cerebrovascular diseases; CAD: Coronary artery excluding MI; MI: Myocardial infarction

Table S7. Hazard ratios (95% CI) for CDV/CEV and mortality risks according to the adherence ≥ 60% vs < 60% (reference) after IPTW matching in the total cohort and sub-cohorts for atypical antipsychotic users.

**Total cohort**	Incident rates in the 5-year period, per 100 person-years (95% CI) after IPTW matching		Hazard ratios (95% CI) (ref.: < 60%)	p-value
	< 60% (n=23,219)	≥ 60% (n=19,431)		
CVD/CEV events				
CAD	9.6 (9.4-9.9)	8.5 (8.2-8.7)	0.88 (0.85-0.92)	<0.0001
Stroke	4.9 (4.7-5.0)	4.6 (4.4-4.8)	0.95 (0.90-1.01)	0.0803
MI	1.7 (1.6-1.8)	1.6 (1.5-1.7)	0.97 (0.89-1.06)	0.4641
All CVD/CEV	14.4 (14.1-14.7)	13.2 (12.9-13.6)	0.93 (0.90-0.96)	<0.0001
All-cause mortality	24.5 (24.2-24.9)	17.6 (17.3-18.0)	0.74 (0.72-0.75)	<0.0001

**Sub-cohort without schizophrenia/dementia**	Incident rates in the 5-year period, per 100 person-years (95% CI) after IPTW matching		Hazard ratios (95% CI) (ref.: < 60%)	p-value
	< 60% (n=18,002)	≥ 60% (n=9,398)		
CVD/CEV events				
CAD	10.4 (10.1-10.7)	9.4 (9.0-9.9)	0.91 (0.86-0.96)	0.0002
Stroke	4.5 (4.3-4.7)	4.5 (4.2-4.7)	0.99 (0.92-1.06)	0.7472
MI	1.7 (1.6-1.8)	1.7 (1.6-1.9)	1.01 (0.90-1.13)	0.8777
All CVD/CEV	14.6 (14.2-14.9)	14.0 (13.5-14.5)	0.97 (0.92-1.01)	0.1239
All-cause mortality	23.4 (23.0-23.8)	15.0 (14.5-15.5)	0.67 (0.65-0.70)	<0.0001

**Sub-cohort of patients with schizophrenia**	Incident rates in the 5-year period, per 100 person-years (95% CI) after IPTW matching		Hazard ratios (95% CI) (ref.: < 60%)	p-value
	< 60% (n=278)	≥ 60% (n=513)		
CVD/CEV events				
CAD	6.1 (4.6-7.5)	7.4 (6.1-8.7)	1.22 (0.90-1.64)	0.2008
Stroke	3.6 (2.5-4.7)	4.8 (3.8-5.9)	1.34 (0.92-1.95)	0.1237
MI	1.2 (0.6-1.8)	1.4 (0.9-1.9)	1.18 (0.62-2.25)	0.6252
All CVD/CEV	9.1 (7.3-10.9)	12.1 (10.4-13.8)	1.33 (1.04-1.70)	0.0233
All-cause mortality	11.8 (9.9-13.8)	10.4 (8.9-11.8)	0.88 (0.71-1.09)	0.2320

**Sub-cohort of patients with dementia**	Incident rates in the 5-year period, per 100 person-years (95% CI) after IPTW matching		Hazard ratios (95% CI) (ref.: < 60%)	p-value
	< 60% (n=9,750)	≥ 60% (n=5,035)		
CVD/CEV events				
CAD	8.3 (7.8-8.8)	7.1 (6.8-7.4)	0.86 (0.80-0.92)	<0.0001
Stroke	5.5 (5.1-5.9)	4.8 (4.6-5.1)	0.88 (0.81-0.97)	0.0062
MI	1.7 (1.5-1.9)	1.5 (1.3-1.6)	0.86 (0.74-1.01)	0.0651
All CVD/CEV	14.1 (13.4-14.7)	12.1 (11.7-12.6)	0.87 (0.82-0.92)	<0.001
All-cause mortality	25.0 (24.3-25.8)	21.9 (21.4-22.5)	0.88 (0.84-0.91)	<0.0001

CVD/CEV: Cardiovascular and cerebrovascular diseases; CAD: Coronary artery excluding MI; MI: Myocardial infarction

Table S8. Hazard ratios (95% CI) for CVD/CEV and mortality risks according to the adherence ≥ 60% vs < 60% (reference) after IPTW matching in the total cohort and sub-cohorts for typical antipsychotic users.

**Total cohort**	Incident rates in the 5-year period, per 100 person-years (95% CI) after IPTW matching		Hazard ratios (95% CI) (ref.: < 60%)	p-value
	< 60% (n=23,219)	≥ 60% (n=19,431)		
CVD/CEV events				
CAD	9.1 (8.9-9.3)	9.7 (8.8-10.8)	1.03 (0.91-1.15)	0.6805
Stroke	4.8 (4.6-4.9)	4.4 (3.7-5.2)	0.88 (0.74-1.04)	0.1405
MI	1.7 (1.6-1.7)	1.7 (1.2-2.1)	0.94 (0.72-1.23)	0.6512
All CVD/CEV	13.9 (13.7-14.1)	14.0 (12.7-15.4)	0.96 (0.87-1.06)	0.3680
All cause mortality	20.2 (19.9-20.4)	76.2 (73.2-79.2)	3.35 (3.22-3.50)	<0.0001

**Sub-cohort without schizophrenia/dementia**	Incident rates in the 5-year period, per 100 person-years (95% CI) after IPTW matching		Hazard ratios (95% CI) (ref.: < 60%)	p-value
	< 60% (n=18,002)	≥ 60% (n=9,398)		
CVD/CEV events				
CAD	10.1 (9.9-10.4)	9.2 (7.8-10.6)	0.89 (0.76-1.03)	0.1129
Stroke	4.5 (4.3-4.6)	3.2 (2.4-4.0)	0.68 (0.53-0.87)	0.0020
MI	1.7 (1.7-1.8)	1.3 (0.8-1.8)	0.71 (0.48-1.04)	0.0758
All CVD/CEV	14.4 (14.2-14.7)	12.0 (10.4-13.6)	0.79 (0.69-0.91)	0.0007
All-cause mortality	19.1 (18.8-19.4)	82.0 (78.1-85.9)	3.58 (3.40-3.76)	<0.0001

**Sub-cohort of patients with schizophrenia**	Incident rates in the 5-year period, per 100 person-years (95% CI) after IPTW matching		Hazard ratios (95% CI) (ref.: < 60%)	p-value
	< 60% (n=278)	≥ 60% (n=513)		
CVD/CEV events				
CAD	6.9 (5.9-7.9)	5.7 (1.4-10.1)	0.82 (0.38-1.78)	0.6196
Stroke	4.4 (3.6-5.2)	3.5 (0.2-6.8)	0.80 (0.31-2.07)	0.6464
MI	1.4 (0.9-1.8)	0.7 (0.0-2.2)	0.54 (0.07-4.14)	0.5506
All CVD/CEV	11.0 (9.7-12.3)	9.8 (4.0-15.6)	0.89 (0.49-1.62)	0.7053
All-cause mortality	10.8 (9.6-12.0)	13.4 (7.1-19.8)	1.25 (0.77-2.02)	0.3695

**Sub-cohort of patients with dementia**	Incident rates in the 5-year period, per 100 person-years (95% CI) after IPTW matching		Hazard ratios (95% CI) (ref.: < 60%)	p-value
	< 60% (n=9,750)	≥ 60% (n=5,035)		
CVD/CEV events				
CAD	7.4 (7.2-7.7)	11.3 (8.9-13.7)	1.45 (1.16-1.80)	0.0009
Stroke	5.1 (4.9-5.3)	5.1 (3.5-6.7)	0.95 (0.69-1.31)	0.7593
MI	1.5 (1.4-1.6)	2.7 (1.6-3.8)	1.66 (1.09-2.55)	0.0195
All CVD/CEV	12.7 (12.4-13.1)	17.0 (14.0-20.1)	1.25 (1.04-1.51)	0.0153
All-cause mortality	22.4 (22.0-22.9)	55.5 (50.4-60.6)	2.45 (2.23-2.69)	<0.0001

CVD/CEV: Cardiovascular and cerebrovascular diseases; CAD: Coronary artery excluding MI; MI: Myocardial infarction

Table S9. Hazard ratios (95% CI) for CVD/CEV and mortality risks according to the adherence ≥ 70% vs < 70% (reference) after IPTW marching according to the adherence level in the total cohort.

	CVD/CEV events		Mortality	
	HR 95% CI	p-value	HR 95% CI	p-value
Total cohort				
Adherence ≥ 70% (ref.: <70%)	0.92 (0.89-0.95)	<0.0001	0.97 (0.95-0.99)	0.0281
Sub-cohort without schizophrenia/dementia				
Adherence ≥ 70% (ref.: <70%)	0.94 (0.90-0.98)	0.0038	0.99 (0.96-1.03)	0.6149
Sub-cohort of patients with schizophrenia				
Adherence ≥ 70% (ref.: <70%)	1.45 (1.13-1.85)	0.0033	1.01 (0.81-1.24)	0.9903
Sub-cohort of patients with dementia				
Adherence ≥ 70% (ref.: <70%)	0.88 (0.83-0.94)	<0.0001	0.99 (0.95-1.03)	0.4847

Table S10. Description of data sources: RAMQ database

The cohort is coming from the Med-Echo administrative databases and the "*Régie de l'Assurance Maladie du Québec*" (RAMQ). The Med-Echo administrative databases, which store data on hospital discharges, and RAMQ databases, which store data on medical services, and public drug plans, and all those databases are managed by the RAMQ.[1-4] The databases were linked using encrypted health insurance numbers. The information from these databases provide a comprehensive picture of the status of hospital admissions, medical services and public drug plans.

Data were collected from the RAMQ and Med-Echo databases, which administer public healthcare insurance programs in the province of Quebec, Canada. The Med-Echo database gathers information on acute care hospitalizations, such as date of admission, length of stay, primary and up to 15 secondary diagnoses. The RAMQ data were extracted from three databases. The beneficiary database lists age, gender, socioeconomic status, and date of death. The medical services file contains claims for all inpatient and ambulatory services and diagnostic codes are classified according to the International Classification of Diseases, 9th Revision (ICD-9). All surgical procedures codes follow the Canadian classification of diagnostic, therapeutic and surgical procedures.[5] The pharmaceutical database provides data on delivered medication in community pharmacies such as the date of filling, name of the drug, dose, quantity, dosage form, and duration of therapy. The RAMQ covers all Quebec residents for the cost of physician visits, hospitalizations and procedures, and 94% of Quebec citizens aged 65 and older for the drug plan.[1,3]

1. Tamblyn, R., Lavoie, G., Petrella, L. & Monette, J. The use of prescription claims databases in pharmacoepidemiological research: the accuracy and comprehensiveness of the prescription claims database in Quebec. *J. Clin. Epidemiol*. **48**, 999-1009 (1995).
2. Eguale, T., Winslade, N., Hanley, J.A., Buckeridge, D.L. & Tamblyn, R. Enhancing pharmacosurveillance with systematic collection of treatment indication in electronic prescribing: a validation study in Canada. *Drug Saf*. **33**, 559-567 (2010).
3. Wilchesky, M., Tamblyn, R.M. & Huang, A. Validation of diagnostic codes within medical services claims. *J. Clin. Epidemiol*. **57**, 131-141 (2004).
4. Tamblyn, R., Reid, T., Mayo, N., McLeod, P. & Churchill-Smith, M. Using medical services claims to assess injuries in the elderly: sensitivity of diagnostic and procedure codes for injury ascertainment. *J. Clin. Epidemiol*. **53**, 183-194 (2000).
5. Régie de l'assurance maladie du Québec. Rapport annuel de gestion 2005-2006. Quebec: Régie de l'assurance maladie du Québec (2006).

ICD-9 and ICD-10, International Classification of Diseases, 9th and 10th Revision; RAMQ, *Régie de l'Assurance Maladie du Québec*.

Table S11. Definition of cardiovascular risk factors or diseases according to ICD-9 and ICD-10 from Med-Echo databases or RAMQ medical service files.

**Diagnosis**	**ICD-9 codes**	**ICD-10 codes**
**Hypertension**	401	I10
**Diabetes**	250.x	E08, E10, E11, E13
**Dyslipidemia**	272	E78
**Coronary artery disease**	410-414	I20-I25
**Myocardial infarction**	410.xx	I21, I22, I23
**Stroke**		
Hemorrhagic stroke intracranial (non-traumatic)	430, 431, 432.x	I60, I61, I62
Ischaemic stroke	433.xx, 434.xx, 436.0, 436.9	I63 except I63.6, I64
Transient ischemic attack	435.x	G45
**Chronic heart failure**	428.0, 428.1, 428.9	I50, I50.1, I50.9
**Atrial fibrillation**	427.3	I48
**Major bleeding**		
**-Intracranial major bleeding**		
Intracranial bleeding	430, 431, 432.x, 852.x, 853.x	I60, I61, I62, S06.3, S06.4, S06.5, S06.6
**-Major gastrointestinal bleeding**		
Upper gastrointestinal bleeding (only using Med-Echo)	456.1, 530.7, 531.0x, 531.2x, 531.4x, 531.6x, 532.0x, 532.2x, 532.4x. 532.6x, 533.0x. 533.2x, 533.4x, 533.6x, 534.0x, 534.2x, 534.4x, 534.6x, 535.1, 578.0	I85.0, K22.6, K25.0, K25.2, K25.4, K25.6, K26.0, K26.2, K26.4, K26.6, K27.0, K27.2, K27.4, K27.6, K28.0, K28.2, K28.4, K28.6, K29.0, K92.0
Upper gastrointestinal bleeding (only using RAMQ)	456.1, 530.7, 531.0x, 531.2x, 531.4x, 531.6x, 532.0x, 532.2x, 532.4x. 532.6x, 533.0x, 533.2x, 533.4x, 533.6x, 534.0x, 534.2x, 534.4x, 534.6x, 535.1, 578.0 RAMQ ICD-9 at an emergency room and procedure endoscopic control of gastric or duodenal bleeding or upper	I85.0, K22.6, K25.0, K25.2, K25.4, K25.6, K26.0, K26.2, K26.4, K26.6, K27.0, K27.2, K27.4, K27.6, K28.0, K28.2, K28.4, K28.6, K29.0, K92.0 RAMQ ICD-9 at an emergency room and procedure endoscopic control of gastric

	gastrointestinal endoscopy including esophagus, stomach, and either the duodenum and/or jejunum as appropriate with control of bleeding, any method (code 00691) within 7 days	or duodenal bleeding or upper gastrointestinal endoscopy including esophagus, stomach, and either the duodenum and/or jejunum as appropriate with control of bleeding, any method (00691) within 7 days
Lower gastrointestinal bleeding	562.02, 562.03, 562.12, 562.13, 569.3x, 569.85, 578.1x, 578.9	K57.11, K57.13, K57.31, K57.33, K62.5, K55.21, K92.1, K92.2
**-Other critical sites of major bleeding**		
Gross hematuria	599.7	R31
Hemoptysis	786.3x	R04.2, R04.89, R04.9
Vitreous hemorrhage	379.23	H43.13
Urogenital bleed	626.2x and 280.0, 285.1 or 285.9	N92.0 and D50.0, D62, D64.9)
Hemarthrosis	719.1x	M25.0x
Hemopericardium	423.0	I31.2
Hemoperitoneum	568.8	K66.1
Hemorrhage not specified	459.0x	R58.0
Acute posthemorrhagic anemia	285.1x	D62
**-Systemic embolism**	444.x, 557.0, 362.31, 362.32, 598.31	I74
Arterial embolism and thrombosis	444.x	I74.0, I74.1, I74.2, I74.3, I74.5, I74.8, I74.9
Ischemic colitis or mesenteric thromboembolism	557.0	K55.0
Retinal artery thromboembolism	362.31, 362.32	H34.1, H34.2
Renal artery thromboembolism	593.81	N28.0
**Peripheral arterial disease**	440 (except 440.0), 441, 443.0, 443.89, 443.9	I70.1 to I70.9, I71, I73.0, I73.89, I73.9
**Chronic kidney disease** (moderate to severe disease)	404.01, 404.03, 404.11, 404.13, 404.91, 404.93, 580.0, 580.4, 581.0, 581.1, 581.2, 581.3, 581.89, 581.9, 582.0, 582.1, 582.2, 582.89, 582.9, 583.0, 583.1, 583.2, 583.4, 583.7, 583.6, 583.89, 583.9, 584.5, 584.6, 584.7, 584.8, 584.9, 585.1, 585.2,	I12, I13, N00, N01, N02, N03, N04, N05, N07, N11, N12, N14, N17, N18, N19

	585.3, 585.4, 585.5, 585.6, 586, 590.0, 590.01, 590.80	
**Schizophrenia**	295.0-295.6, 295.8-295.9	F20
**Dementia**	46.1, 331.0, 331,1, 331.5, 290.x, 294.x and/or medication code (donepezil, rivastigmine, galantamine, memantine)).	G30, F00, F01, F02, F03 and/or medication code (donepezil, rivastigmine, galantamine, memantine)).

ICD-9, International Classification of Diseases, 9th Revision; ICD-10, International Classification of Diseases, 10th Revision;
RAMQ : Régie de l'assurance maladie du Québec.
Med-Echo: Administrative databases of hospital discharge reports

.

Table S12. Olanzapine equivalents based Defined Daily Dose (DDDs)

**Neuroleptics**	**Conversion in olanzapine (mg)**
Amisulpride	dose/40[a]
Aripiprazole	dose/1,5 [a]
Chlorpromazine	dose/30[a]
Clozapine	dose/30[a]
Flupenthixol	dose/0,6[a]
Fluphenazine	dose[a]
Haloperidol	dose/0,8[a]
Loxapine	dose/10[a]
Lurasidone	dose/6[a]
Methotrimeprazine	dose/0,067[b]
Olanzapine	dose[a]
Paliperidone	dose/0.6
Perphenazine	dose/3[a]
Pimozide	dose/0,4[a]
Prochlorperazine	dose/10a
Quetiapine	dose/40a
Risperidone	dose/0,5a
Trifluoperazine	dose/2a
Ziprasidone	dose/8a
Zuclopenthixol	dose/3a

a : Stefan Leucht - Dose Equivalents for Antipsychotic Drugs: The DDD Method
b : David M. Gardner - International Consensus Study of Antipsychotic Dosing
